# Supplementary material for: Adherence to Covid-19 mitigation measures and its associated factors among health care workers at referral hospitals in Amhara regional state of Ethiopia
Source: PLoS One. 2022 Aug 5;17(8):e0272570. doi: 10.1371/journal.pone.0272570 (PMC9355263; doi:10.1371/journal.pone.0272570)
Supplement: S1 File — (DOCX) [file pone.0272570.s002.docx]

**Adherence to Covid-19 mitigation measures**

Greetings!

This link is sent to you to collect information about “Adherence to Covid-19 mitigation measures and its associated factors among healthcare workers at Referral Hospitals in Amhara Regional State of Ethiopia”. Your participation in this research is entirely voluntary. It is your choice whether to participate or not. If you choose not participate in this research, it has no effect on you or your work. No one, but the researcher will be able to see the information about you that is collected during the research. The findings of this research may benefit health care providers, patients and the community at large. There is no side effect and known risk related with this kind of research so far. Your role in the success of the research is important and it will take a maximum of 20 minutes of participation. I think you have understood the issues in detail. As I told you the survey has no risk and it is confidential.

Are you willing to participate in this study?

Yes No

**If yes, continue**

**Investigators**: Agazhe Aemro, Beletech Fentie, Mulugeta Wassie

**Structured Questionnaire**

1. Age in completed years? _____________________
2. Sex?
3. Male B. Male
4. What is your higher Educational Level?
5. Diploma
6. Degree
7. MSc/MPH
8. Specialist
9. Ph.D
10. Others specify ------------------
11. Your marital status?
12. Single
13. Married
14. Separated/Divorced
15. Widowed
16. What is your Religion?
17. Orthodox
18. Muslim
19. Catholic
20. Protestant
21. Others specify -------------------
22. Average household monthly income in Ethiopian Birr? ---------------------
23. Number of residents in the household (household number)? --------------
24. Do you use public transportation during the pandemic?
25. Yes
26. No
27. Have you school age children?
28. Yes
29. No
30. Is there a household member age 60 years and above?
31. Yes
32. No
33. Are you active smoker?
34. Yes
35. No
36. Are you fully compliance with social isolation during the Covid-19 pandemic?
37. Yes
38. No
39. Have you wear mask every time you leave home and never or hardly ever remove it from the face, during the Covid-19 pandemic?
40. Yes
41. No
42. Did you wash your hand with soap at least six times per day during the Covid-19 pandemic?
43. Yes
44. No
45. Are you fully compliance with Physical distancing (>= 2 meter) during the Covid-19 pandemic?
46. Yes
47. No
48. Have you received suspected Covid-19 medical diagnosis?
49. Yes
50. No
51. Have you undergone a Covid-19 diagnostic test?
52. Yes
53. No
54. How you perceive your health status?
55. Very good
56. Good
57. Bad
58. Very bad
59. Which pre-existing medical condition you had? (you can select more than one answer)
60. None
61. Asthma
62. Diabetes mellitus
63. Heart disease
64. Hypertension
65. HIV/AIDS
66. Chronic kidney disease
67. Cancer
68. Others specify --------------------
69. Have you a known pre-existing autoimmune problem or taking steroid therapy like, Prednisolone?
70. Yes
71. No
72. What is your level of confidence in the capacity of health services to respond to Covid-19 pandemic?
73. Very confident
74. Confident
75. Not very confident
76. Not confident
77. What is your view regarding the information provided by the health Authorities?
78. Clear and understandable
79. Unclear and confusing
80. Inconsistent and contradictory
81. What is your perception on the adequacy of measures implemented by the Ethiopian government?
82. Very adequate
83. Adequate
84. Not Very adequate
85. Not adequate
86. How you perceive yourself regarding risk to get Covid-19 infection?
87. High risk
88. Moderate risk
89. Low risk
90. No risk
91. Not sure
92. How you perceive yourself regarding risk to develop sever disease following Covid-19 infection?
93. High risk
94. Moderate risk
95. Low risk
96. No risk
97. Not sure
98. By the time you get a chance for Covid-19 vaccine (AstraZeneca), will you take the vaccine without any refusal?
99. Yes
100. No
101. How much you are confident in the efficacy and safety of Covid-19 vaccine (AstraZeneca)?
102. Very confident
103. Confident
104. Not very confident
105. Not confident
106. Do you think that the side effects of AstraZeneca vaccine will be tolerable?
107. Tolerable
108. Not tolerable
109. Not sure

Thank you!!
