# Supplementary material for: Adherence to Covid-19 mitigation measures and its associated factors among health care workers at referral hospitals in Amhara regional state of Ethiopia
Source: PLoS One. 2022 Aug 5;17(8):e0272570. doi: 10.1371/journal.pone.0272570 (PMC9355263; doi:10.1371/journal.pone.0272570)

PLOS ONE

Adherence of Covid-19 mitigation measures and its associated factors among health care workers at Referral Hospitals in Amhara Regional state of Ethiopia.

--Manuscript Draft--

**Manuscript Number:**	PONE-D-21-38230
**Article Type:**	Research Article
**Full Title:**	Adherence of Covid-19 mitigation measures and its associated factors among health care workers at Referral Hospitals in Amhara Regional state of Ethiopia.
**Short Title:**	Adherence of Covid-19 mitigation measures and its associated factors among health care workers
**Corresponding Author:**	Beletech Fentie<br>University of Gondar<br>Gondar, ETHIOPIA
**Keywords:**	Adherence, COVID-19, Mitigation measures, Amhara regional state
**Abstract:**	Introduction: With fragile health care systems, sub-Saharan Africa countries like Ethiopia are facing a complex epidemic, and become difficult to control the noble coronavirus. The use of COVID-19 preventive measures is strongly recommended. This study aimed to assess the adherence of COVID-19 mitigation measures and associated factors among health care workers. Methods and materials: A facility-based cross-sectional study was conducted from May 15 to June 10; 2021. It was a web-based study using an online questionnaire. It was conducted at referral hospitals in the Amhara regional state of Ethiopia among health care workers. STATA 14.2 was used for data analysis. Variables with a p-value<0.05 at 95% confidence level in multivariable analysis were declared as statistically significant using binary logistic regression. Result: Adherence to COVID-19 mitigation measures was 50.24% in the current study. The odd of adherence of participants with a monthly income of ≥12801birr was 15% whereas the odds of adherence of participants who hesitate to take the COVID 19 vaccine was 10% as compared to those who don't hesitate. Participants who had undergone COVID-19 tests adhered 6.64 times more than their counterparts. Those who believe adequate measurements are taken by the government adhered 4.6 times more than those who believe not adequate. Participants who believe as no risk of severe disease adhered 16% compared to those with fear of severe disease. Presence of households aged >60years adhered about 7.9 times more than with no households aged>60. Participants suspected of COVID-19 diagnosis adhered 5.7 times more than those not suspected. Conclusion: This study found lower adherence to COVID-19 mitigation measures. It is better to boost the practice of health care workers on the prevention methods of the COVID-19 pandemic in the current study setting. Keywords: Adherence, COVID-19, Mitigation measures, Amhara regional state
**Order of Authors:**	Agazhe Aemro
	Beletech Fentie
	Mulugeta Wassie
**Additional Information:**	
**Question**	**Response**
**Financial Disclosure**<br><br>Enter a financial disclosure statement that describes the sources of funding for the work included in this submission. Review the submission guidelines for detailed requirements. View published research articles from *PLOS ONE* for specific	The author(s) received no specific funding for this work

Powered by Editorial Manager® and ProduXion Manager® from Aries Systems Corporation

examples.<br><br>This statement is required for submission and **will appear in the published article** if the submission is accepted. Please make sure it is accurate.<br><br>**Unfunded studies**<br>Enter: *The author(s) received no specific funding for this work.*<br><br>**Funded studies**<br>Enter a statement with the following details:<br>• Initials of the authors who received each award<br>• Grant numbers awarded to each author<br>• The full name of each funder<br>• URL of each funder website<br>• Did the sponsors or funders play any role in the study design, data collection and analysis, decision to publish, or preparation of the manuscript?<br>• **NO** - Include this sentence at the end of your statement: *The funders had no role in study design, data collection and analysis, decision to publish, or preparation of the manuscript.*<br>• **YES** - Specify the role(s) played.<br><br>* typeset	
**Competing Interests**<br><br>Use the instructions below to enter a competing interest statement for this submission. On behalf of all authors, disclose any competing interests that could be perceived to bias this work—acknowledging all financial support and any other relevant financial or non-financial competing interests.<br><br>This statement is required for submission and **will appear in the published article** if the submission is accepted. Please make sure it is accurate and that any funding sources listed in your Funding Information later in the submission form are also declared in your Financial Disclosure statement.	The authors have declared that no competing interests exist

View published research articles from *PLOS ONE* for specific examples.<br><br>**NO authors have competing interests**<br><br>Enter: *The authors have declared that no competing interests exist.*<br><br>**Authors with competing interests**<br><br>Enter competing interest details beginning with this statement:<br><br>*I have read the journal's policy and the authors of this manuscript have the following competing interests: [insert competing interests here]*<br><br>* typeset	
**Ethics Statement**<br><br>Enter an ethics statement for this submission. This statement is required if the study involved:<br><br>• Human participants<br>• Human specimens or tissue<br>• Vertebrate animals or cephalopods<br>• Vertebrate embryos or tissues<br>• Field research<br><br>Write "N/A" if the submission does not require an ethics statement.<br><br>General guidance is provided below. Consult the submission guidelines for detailed instructions. **Make sure that all information entered here is included in the Methods section of the manuscript.**	This study was approved by the institutional review board (IRB) of the University of Gondar. Respondents were informed that their participation was voluntary and their confidentiality was maintained by avoiding registration of personal identifiers like names on the questionnaire and also, no raw data was given to anyone other than the investigator. In addition, the raw data is secured by a strong computer password.

**Format for specific study types**<br><br>**Human Subject Research (involving human participants and/or tissue)**<br>• Give the name of the institutional review board or ethics committee that approved the study<br>• Include the approval number and/or a statement indicating approval of this research<br>• Indicate the form of consent obtained (written/oral) or the reason that consent was not obtained (e.g. the data were analyzed anonymously)<br><br>**Animal Research (involving vertebrate animals, embryos or tissues)**<br>• Provide the name of the Institutional Animal Care and Use Committee (IACUC) or other relevant ethics board that reviewed the study protocol, and indicate whether they approved this research or granted a formal waiver of ethical approval<br>• Include an approval number if one was obtained<br>• If the study involved *non-human primates*, add *additional details* about animal welfare and steps taken to ameliorate suffering<br>• If anesthesia, euthanasia, or any kind of animal sacrifice is part of the study, include briefly which substances and/or methods were applied<br><br>**Field Research**<br><br>Include the following details if this study involves the collection of plant, animal, or other materials from a natural setting:<br>• Field permit number<br>• Name of the institution or relevant body that granted permission	
**Data Availability**<br><br>Authors are required to make all data underlying the findings described fully available, without restriction, and from the time of publication. PLOS allows rare exceptions to address legal and ethical concerns. See the PLOS Data Policy and FAQ for detailed information.	Yes - all data are fully available without restriction

A Data Availability Statement describing where the data can be found is required at submission. Your answers to this question constitute the Data Availability Statement and **will be published in the article**, if accepted.<br><br>**Important:** Stating 'data available on request from the author' is not sufficient. If your data are only available upon request, select 'No' for the first question and explain your exceptional situation in the text box.<br><br>Do the authors confirm that all data underlying the findings described in their manuscript are fully available without restriction?	
**Describe where the data may be found in full sentences. If you are copying our sample text, replace any instances of XXX with the appropriate details.**<br><br>• If the data are **held or will be held in a public repository**, include URLs, accession numbers or DOIs. If this information will only be available after acceptance, indicate this by ticking the box below. For example: *All XXX files are available from the XXX database (accession number(s) XXX, XXX.)*.<br>• If the data are all contained **within the manuscript and/or Supporting Information files**, enter the following: *All relevant data are within the manuscript and its Supporting Information files.*<br>• If neither of these applies but you are able to provide **details of access elsewhere**, with or without limitations, please do so. For example:<br><br>*Data cannot be shared publicly because of [XXX]. Data are available from the XXX Institutional Data Access / Ethics Committee (contact via XXX) for researchers who meet the criteria for access to confidential data.*<br><br>*The data underlying the results presented in the study are available from (include the name of the third party*	The datasets used in the current study are available from the corresponding author

and contact information or URL).<br>• This text is appropriate if the data are owned by a third party and authors do not have permission to share the data.<br><br>* typeset	
Additional data availability information:	

Manuscript Click here to access/download;Manuscript;Manuscript.docx

Adherence of Covid-19 mitigation measures and its associated factors among health care workers at Referral Hospitals in Amhara Regional state of Ethiopia.

Adherence of Covid-19 mitigation measures and its associated factors among health care workers

Agazhe Aemro[1], Beletech Fentie[2*], Mulugeta Wassie[1]

1 Department of Medical Nursing, College of Medicine and Health Sciences, University of Gondar, Ethiopia

2 Department Pediatrics and Child Health Nursing, College of Medicine and Health Sciences, University of Gondar, Ethiopia

Email addresses:

AA:agazhe1049@gmail.com

BF:beletechfentie@gmail.com

MW:mulugeta2113@gmail.com

Corresponding to Beletech Fentie, beletechfentie@gmail.com

Abstract

Introduction: With fragile health care systems, sub-Saharan Africa countries like Ethiopia are facing a complex epidemic, and become difficult to control the noble coronavirus. The use of COVID-19 preventive measures is strongly recommended. This study aimed to assess the adherence of COVID-19 mitigation measures and associated factors among health care workers.

Methods and materials: A facility-based cross-sectional study was conducted from May 15 to June 10; 2021. It was a web-based study using an online questionnaire. It was conducted at referral hospitals in the Amhara regional state of Ethiopia among health care workers. STATA 14.2 was used for data analysis. Variables with a p-value<0.05 at 95% confidence level in multivariable analysis were declared as statistically significant using binary logistic regression.

Result: Adherence to COVID-19 mitigation measures was 50.24% in the current study. The odd of adherence of participants with a monthly income of ≥12801birr was 15% whereas the odds of adherence of participants who hesitate to take the COVID 19 vaccine was 10% as compared to those who don't hesitate. Participants who had undergone COVID-19 tests adhered 6.64 times more than their counterparts. Those who believe adequate measurements are taken by the government adhered 4.6 times more than those who believe not adequate. Participants who believe as no risk of severe disease adhered 16% compared to those with fear of severe disease. Presence of households aged >60years adhered about 7.9 times more than with no households aged>60. Participants suspected of COVID-19 diagnosis adhered 5.7 times more than those not suspected.

Conclusion: This study found lower adherence to COVID-19 mitigation measures. It is better to boost the practice of health care workers on the prevention methods of the COVID-19 pandemic in the current study setting.

Keywords: Adherence, COVID-19, Mitigation measures, Amhara regional state

Introduction

The COVID-19 pandemic entered Africa by the end of February 2020 after it was declared a Public Health Emergency of International Concern by the world health organization[1]. With fragile health care systems, sub-Saharan Africa like Ethiopia is facing a complex COVID-19 epidemic and it becomes a difficult task to control the virus reservoir, from where the virus may be reintroduced to other regions [2].

Globally, COVID-19 affected over 119.7 million confirmed cases and 2.6 million deaths[3] whereas in Africa over 4 million confirmed cases and over 107 thousand deaths have been reported [4]. Considering its pandemic and absence of effective treatment, authorities across the world have designed various mitigation measures to combat the spread of COVID-19 [5, 6]. To control the pandemic transmission, WHO recommends minimizing contact, early detection and isolation of cases, personal and material hygiene measures [6, 7].

As part of these measures, the use of face masks, hand washing, physical distancing, cough etiquette, and avoidance of crowded places are strongly recommended [7]. Even though adherence to preventive measures is the only means to tackle the disease, reluctance to do so has been reported to be a major problem everywhere [8].

Health care professionals are experiencing higher workload, psychological distress, shortage of quality personal protective equipment, social exclusion, lack of incentives, absence of coordination, and proper management during their service[9].

The prevalence of good adherence towards COVID-19 was 51.04% and 8.3% in different Ethiopian studies conducted in the general community [10, 11]. As far our search, there is no

research conducted among health care workers in the current study setting and it is also true in the country as large. Therefore, this study intended to assess the adherence of COVID-19 mitigation measures and their associated factors among health care providers in the Amhara region regional state of Ethiopia.

Methods and Materials

Study design, period and Setting

A facility-based cross-sectional study was conducted from May 15 to June 10; 2021. It was a Web-based anonymous study using an online questionnaire. The study was conducted at referral hospitals in the Amhara regional state. According to the Amhara national regional health bureau annual performance report, the region has 81 hospitals, 858 health centers, and 3560 health posts. Among the hospitals in the region, the University of Gondar, Dessie, Felege-Hiwot, Tibebe-Ghion, Debre-Markos, Waldiya, Debre Tabor, and Debebirhan are referral hospitals. The health care professionals working in these hospitals are estimated to be 4,000 [12, 13].

Study participants

Telegram and email (the most popular social media platforms in Ethiopia) were used to promote and circulate the survey link to the participants. Data collectors in each hospital were asked to distribute the survey link to the randomly selected contacts in each hospital. The participants were informed that their participation was based on voluntariness, and consent was implied through their completion of the questionnaire. The respondents working during the data collection period were included in the current study.

Sample Size Determination

The sample size was determined using the single population proportion formula taking the proportion of compliance to the COVID19 preventive measures 22%[14], 95% confidence interval, and 4% marginal error. After adding a 5% non-response rate, the final sample size was ~433.

Sampling Procedure

There are about 4,000 health care workers in Amhara regional state referral hospitals(906 in Gondar hospital, 320 in Debre tabor hospital,255 in Tibebe Ghione hospital,917 in Felege Hiot hospital,430 in Debre Markos Hospital,604 in Dessie hospital,300 in Woldiya hospital and 270 in Debre Berhan hospital). The entire sample size was first allocated proportionally to those eight referral hospitals. Then, the link of the questionnaire was given to the data collectors and forwarded to randomly selected health care workers of respected hospitals, using e-mail or telegram. The link was forwarded to each hospital's data collector to avoid coverage bias and to be representative.

Operational Definitions

Good adherence of COVID-19 mitigation measures: Adherence in the current study was measured as participants who adhered (responded "yes") to all of the three basic preventive measures (Wearing a mask, keeping physical distancing of a minimum of 2 meters, and hand washing a minimum of ≥6 times/ day) and measured 'Yes' or 'No' answers to the questions.

Health Care worker (HCW): Any member of the health care unit that includes medical doctors, pharmacists, physiotherapists, midwifery, laboratory technologists, nursing professions, or any other person in the course of his or her professional activities who may prescribe, administer, or dispense a medicinal product to an end-user [15].

Vaccine Hesitancy: World Health Organization (WHO) declared vaccine hesitancy as "the reluctance or refusal to vaccinate despite the availability of vaccines" [16].

Perceived susceptibility/risk of getting COVID-infection: Refers to a participant's subjective perception of the risk of acquiring COVID-19 and is measured as High, Moderate, Low, No risk, or not sure [17].

Perceived severity/risk of developing the severe disease: Refers to a person's subjective perception of the seriousness of contracting COVID-19 and measured as High, Moderate, Low, No risk, or not sure [17].

Data Processing and Analysis

The responses from the participants were downloaded in Excel using Google Forms. The data were checked for completeness and consistency, then compiled and coded. After that, it was exported to STATA version 14.2 statistical software for analysis. Descriptive statistics were used to summarize the results and presented using tables. Binary logistic regression was used to identify factors that affect the outcome variable. Variables with a p-value less than or equal to 0.2 in the bi-variable analysis were candidates for multivariable analysis. The multivariable analysis was used to declare statistical significance at a p-value of <0.05. Lastly, variables with a p-value<0.05 at a 95% confidence level in multivariable analysis were declared as statistically significant.

Data Quality Assurance

The web-based self-administered questionnaire was pretested by taking 5% of the sample size before the actual data collection period. Afterward, the pretests, amendments to the tool, like formatting were corrected. The tool was first developed in the English language and was translated into the local language (Amharic) with back translation to English to check its

consistency. Moreover, Cronbach's alpha value was calculated to check the tools' reliability and the value of an item score was 0.892.

Ethics approval and consent to participate

This study was approved by the institutional review board (IRB) of the University of Gondar. Respondents were informed that their participation was voluntary and their confidentiality was maintained by avoiding registration of personal identifiers like names on the questionnaire and also, no raw data was given to anyone other than the investigator. In addition, the raw data is secured by a strong computer password.

Results

Socio-demographic characteristics of study participants

From the total 433 samples, 418 participants completed the questionnaire that yielded a 96.5% response rate. The mean age of study participants was 29.95 in the current study. More than half of the participants were under the age category of 26-30 years and nearly two-thirds were males. About 54% were married, 55% BSc and below educational level. The majority of the study participants have a monthly income in the category of 6991-12800 birr. Based on family size, 53.35% have less than or equal to 2 and nearly one thirds (31.58%) have children with school-age (**Table 1).**

Table 1: Socio demographic characteristics of study participants (N=418)

COVID-19 related characteristics of study participants

Nearly two-thirds (63.64%) of the participants were socially isolated because of their profession. About 59% underwent the COVID test and 44% were confident in health care services delivered on their institution whereas 45.69% got unclear information by health authorities related to the

COVID-19 pandemic. Only100 (23.92%) believe measurements taken by the national government related to COVID-19 preventive measures are adequate. More than half (52.39%) of participants reported that they are at higher risk of COVID-19 infection but 53.35% believe they are at low risk to develop the severe disease if infected with the coronavirus. Nearly two-thirds (63.64%) had good compliance on social isolation if suspected to COVID-19 whereas 57.89% were suspected of COVID-19 diagnosis. About 55% perceive that their health status was very good. Only 3.83% have autoimmune diseases taking steroidal drugs. Nearly 54% of the participants were willing to take the COVID-19 vaccine but about 19% are confident in the current vaccine (**Table 2).**

Table 2: COVID-19 related characteristics of the study participants (N=418)

Adherence towards COVID-19 mitigation measures

The Adherence towards COVID-19 mitigation measures among health care workers in the current study was 50.24[95%CI (45.44- 55.04)]. Adherence to COVID-19 measures was 71.29%,73.21%, and 56.94% for wearing a mask, washing hands ≥6 times per day based on WHO handwashing rules, and physical distancing of at least 2 meters respectively**(Fig 1).**

Figure 1: Distribution of Adherence of COVID-19 mitigation measures among health care workers in referral hospitals of Amhara regional state of Ethiopia

Factors associated with adherence of COVID-19 mitigation measures

Binary logistic regression was employed to identify independent factors that can affect the outcome variable. In bivariable analysis, monthly income, hesitancy to take COVID-19 vaccine, age, marital status, undergone COVID-19 test, the information given by health authority, measures taken by the national government, the risk to get COVID-19 disease, risk of severe

COVID-19 disease, household age >60 years, suspected to COVID-19 infection, Comorbidity and Confident on the current COVID-19 vaccine were associated with the outcome variable.

But in multivariable analysis, monthly income, hesitancy to take COVID-19 vaccine, age, undergone COVID-19 test, measures taken by the national government, household aged >60 years, and suspected to COVID-19 infection were statistically significant variables that affected adherence of COVID-19 mitigation measures.

Study participants with a monthly income of ≥12801birr adhered to COVID-19 measures 15% taking monthly income of ≤6990 as reference [AOR=0.15, 95%CI (0.02-0.92)]. Participants who hesitate to take COVID 19 vaccine adhered 10% [AOR=0.10, 95%CI (0.04-0.25)] as compared to those who don't hesitate. Those participants with the age group of 26-30 years adhered to mitigation measures 9% [AOR=0.09, 95%CI (0.02-0.39)] compared to age groups <26 years. Study participants who underwent the COVID-19 test adhered to about 6.6[AOR=6.64, 95%CI (3.10-14.22)] times more than those who didn't undergo the test. Participants who believe adequate measurements are taken by the government adhered to about 4.6 [AOR =4.60, 95%CI (1.66-12.78)] times more than those who believe measurements are not adequate. Participants who believe with no risk of severe COVID-19 disease adhered 16% [AOR=0.16, 95%CI (0.06-.46)] as compared to those with fear of severe COVID-19 disease. Participants who have households aged >60 years adhered about 7.9[AOR =7.94, 95%CI (3.14-20.04)] times more than those with no households aged>60 years and those participants suspected to COVID-19 diagnosis adhered to mitigation measures about 5.7 [AOR=5.74, 95%CI (1.81-18.16)] times more than those who didn't suspect (**Table 3).**

Table 3: Factors associated with adherence of COVID-19 mitigation measures among health care workers in referral hospitals of Amhara regional state of Ethiopia (N=418)

Discussion

The current study aimed to determine adherence to COVID-19 mitigation measures and their associated factors among health care workers in the Amhara regional state of Ethiopia. The adherence of COVID-19 mitigation measures among the participants was 50.24%. The highest adherence (73.21%) was reported for handwashing whereas the lowest (56.94%) was reported for physical distancing.

The current finding of adherence was lower than the study conducted in Saudi Arabia (82%), the United Kingdom(80%), and the Kingdom of Saudi Arabia(80.9%) [18-20]. The possible reasons for this difference might be the countries' policy to prevent the pandemic, the monthly income difference of the study participants which might affect buying abilities of face masks, the data collection period differences in which all the studies conducted before the current study when vaccines were not found. But the current finding is more than the studies conducted in Western Ethiopia (22%) and southeast Ethiopia (21.6 %) [14, 21]. The possible justification of the differences of the findings might be differences in COVID-19 prevention policies of the respected health institutions in the specified regions even though they are found in the same country.

Deferent independent variables in the current study affected the outcome variable. Monthly income, vaccine hesitancy, age, undergone COVID19 test, measurements taken by the national government, perception of the severity of the disease, presence of households with age>60 years and suspected to COVID-19 diagnosis significantly affected the adherence of COVID-19 measures in different directions.

Study participants in the current study with a monthly income of ≥12801birr adhered to COVID-19 measures less than those with a monthly income of ≤6990 birrs. This might be participants

with low monthly income could use public transportation which might increase fear to acquire COVID-19 infection and cause them to adhere more[22, 23]. Participants who hesitate to take COVID -19 vaccines adhered lower than those who are volunteers to take the vaccine. The possible reason could be those who hesitate to take the vaccine might be individuals that believe as COVID -19 is not a severe disease and even there is no such disease [24-26].

Study participants with age groups of 26-30 years adhered to mitigation measures lower than those with age groups of <26 years. This would be younger professionals might abide by mitigation measures more than elders due to negligence [27].

The experienced COVID-19 test increased the participants' adherence to the current study. This might be the participants who believe the existence of the pandemic is high and resulted to undergo COVID-19 test and consequently adhered to the mitigation measures than those who didn’t experience the COVID-19 test[1, 28]. Similarly, study participants who think that adequate measurements are taken by the national government adhered to mitigation measures more than those who think not taking adequate measurements. This could be those thinking the national government is taking adequate measurement trusted the national policies related to the pandemic and consequently adhered more [29, 30].

Study participants who perceived the severity of the disease as High adhered more than those who perceived no risk on COVID-19 mitigation measures. Naturally, those who perceive the disease as severe are more committed to preventing it[31]. Participants who have households aged>60 years adhered more than those with no. This might be because individuals with age >60 years are at the risk of getting severe complications of the COVID -19 like death[32]. Therefore,

those participants with households of age >60 years adhered more to prevent such complications of their households.

Another factor that increased the adherence to theCOVID-19 mitigation measures was suspected of COVID-19 infection. This might be as those suspected of the disease would not be allowed to enter the working area and consequently adhere to the preventive measures[33].

Conclusion

This study found lower adherence to COVID-19 mitigation measures among health care workers. Greater monthly income, hesitate to take the vaccine and older age decreased the adherence whereas undergone COVID-19 test, adequate measurement by the government, believing severity of the disease, households with age >60 years and suspected to COVID-19 diagnosis increased the adherence of mitigation measures. It is better to boost the practice of health care workers on the prevention methods of the COVID-19 pandemic in the current study setting since the adherence of the mitigation measures is lower than the recommended.

Limitations of the study

Since this study is cross-sectional, it shares the limitations of a cross-sectional study design. Social desirability bias could be introduced through study participants since the data collection technique was self-administered. To avoid the mentioned bias, the authors recommend doing further investigation using observational checklists.

Acknowledgment

The authors would like to acknowledge the healthcare workers for their collaboration during the data collection. Our gratitude also goes to data collectors in each hospital. Last but not least, we would like to pass our thanks to the University of Gondar for providing ethical clearance to conduct this study.

Authors' Contribution: AA worked on conception, designing the study, supervising the data collectors, interpreting the result, and preparing the manuscript. BF and MW worked on the conception, analysis, and interpretation of the results and wrote the manuscript. All authors reviewed the results and approved the final version of the manuscript

Financial support

The authors were not funded for this work

Ethics approval and consent to participate

This study was approved by the institutional review board (IRB) of the University of Gondar. Respondents were informed that their participation was voluntary and their confidentiality was maintained by avoiding registration of personal identifiers like names on the questionnaire and also, no raw data was given to anyone other than the investigator. In addition, the raw data is secured by a strong computer password.

Consent for publication.

Not applicable

Conflict of interest

None

Availability of data and materials

The datasets used in the current study are available from the corresponding author.

References

1. Shewasinad Yehualashet, S., et al., *Predictors of adherence to COVID-19 prevention measure among communities in North Shoa Zone, Ethiopia based on health belief model: A cross-sectional study*. PLoS One, 2021. **16**(1): p. e0246006.
2. World Health Organization, *Statement on the second meeting of the International Health Regulations (2005) Emergency Committee regarding the outbreak of novel coronavirus (2019-nCoV)*. 2020.
3. Wondimu, W. and B. Girma, *Challenges and silver linings of COVID-19 in Ethiopia–a short review*. Journal of multidisciplinary healthcare, 2020. **13**: p. 917.

4. Tesfaye, Z.T., et al., *COVID-19-related knowledge, attitude and practice among hospital and community pharmacists in Addis Ababa, Ethiopia.* Integrated Pharmacy Research & Practice, 2020. **9**: p. 105.
5. Azene, Z.N., et al., *Adherence towards COVID-19 mitigation measures and its associated factors among Gondar City residents: A community-based cross-sectional study in Northwest Ethiopia.* PloS one, 2020. **15**(12): p. e0244265.
6. Organization, W.H., *Strengthening preparedness for COVID-19 in cities and urban settings.* 2020.
7. Amgain, K., et al., *Myths versus truths regarding the novel coronavirus disease (COVID-2019) outbreak.* Journal of Karnali Academy of Health Sciences, 2020. **3**(1).
8. Abeya, S.G., et al., *Adherence to COVID-19 preventive measures and associated factors in Oromia regional state of Ethiopia.* PloS one, 2021. **16**(10): p. e0257373.
9. Razu, S.R., et al., *Challenges faced by healthcare professionals during the COVID-19 pandemic: a qualitative inquiry from Bangladesh.* Frontiers in public health, 2021: p. 1024.
10. Azene, Z.N., et al., *Adherence towards COVID-19 mitigation measures and its associated factors among Gondar City residents: A community-based cross-sectional study in Northwest Ethiopia.* 2020. **15**(12): p. e0244265.
11. Abeya, S.G., et al., *Adherence to COVID-19 preventive measures and associated factors in Oromia regional state of Ethiopia.* PLoS One, 2021. **16**(10): p. e0257373.
12. Mekonen, E.G., M.H. Gebrie, and S.M. Jemberie, *Magnitude and associated factors of medication administration error among nurses working in Amhara Region Referral Hospitals, Northwest Ethiopia.* Journal of Drug Assessment, 2020. **9**(1): p. 151-158.
13. Getie, A., A. Wondmieneh, and T. Gebremeskel, *Implementation of nursing process among nurses at Woldia Referral hospital, Northern Ethiopia: An institution-based cross-sectional study.* 2021.
14. Etafa, W., et al., *Healthcare workers' compliance and its potential determinants to prevent COVID-19 in public hospitals in Western Ethiopia.* BMC infectious diseases, 2021. **21**(1): p. 1-8.
15. Walle, Z., et al., *COVID-19 Prevention Practices and Determinant Factors Among Healthcare Professionals Working in Hospitals of South Gondar Zone, Northwestern Ethiopia.* Journal of Multidisciplinary Healthcare, 2021. **14**: p. 2287-2298.
16. WHO. *WHO'S top 10 threats to global health in 2019 - ANMJ.* 2021 [cited 2021 August 9]; Available from: https://anmj.org.au/whos-top-10-threats-to-global-health-in-2019/.
17. Handebo, S., et al., *Determinant of intention to receive COVID-19 vaccine among school teachers in Gondar City, Northwest Ethiopia.* Plos one, 2021. **16**(6): p. e0253499.
18. Albeladi, F.I., et al., *Level of Adherence to COVID-19 Preventive Measures Among Health Care Workers in Saudi Arabia.* Cureus, 2021. **13**(6).
19. Smith, L.E., et al., *Adherence to protective measures among health care workers in the UK; a cross-sectional study.* medRxiv, 2020.
20. Al Khathlan, N. and B.K. Padhi, *Adherence to COVID-19 Appropriate Behavior Among Respiratory Therapists: A Cross-Sectional Study in the Kingdom of Saudi Arabia.* Frontiers in Public Health, 2021. **9**.
21. Zenbaba, D., et al., *Compliance towards infection prevention measures among health professionals in public hospitals, southeast Ethiopia: a cross-sectional study with*

implications of COVID-19 prevention. Tropical Medicine and Health, 2021. **49**(1): p. 1-11.
22. Richert, J., I. Martin, and S. Schrader, *Beyond the immediate crisis: The SARS-CoV-2 pandemic and public transport strategy*. 2020, Berlin: Mobility Institute Berlin.
23. Gkiotsalitis, K. and O. Cats, *Public transport planning adaption under the COVID-19 pandemic crisis: a literature review of research needs and directions.* Transport Reviews, 2021. **41**(3): p. 374-392.
24. Alper, S., F. Bayrak, and O. Yilmaz, *Psychological correlates of COVID-19 conspiracy beliefs and preventive measures: Evidence from Turkey.* Current Psychology, 2020: p. 1-10.
25. Stoica, C.A. and R. Umbreș, *Suspicious minds in times of crisis: determinants of Romanians' beliefs in COVID-19 conspiracy theories.* European Societies, 2021. **23**(sup1): p. S246-S261.
26. Pennycook, G., et al., *Fighting COVID-19 misinformation on social media: Experimental evidence for a scalable accuracy-nudge intervention.* Psychological science, 2020. **31**(7): p. 770-780.
27. Health and C.P. Council, *Professionalism in healthcare professionals*. 2014, Health and Care Professions Council London.
28. Asfaw, L.S., S.Y. Ayanto, and Y.H. Aweke, *Health-seeking behavior and associated factors among community in Southern Ethiopia: Community based cross-sectional study guided by Health belief model.* BioRxiv, 2018: p. 388769.
29. Ning, Y., R. Ren, and G. Nkengurutse, *China's model to combat the COVID-19 epidemic: a public health emergency governance approach.* Global health research and policy, 2020. **5**(1): p. 1-4.
30. Ha, B.T.T., et al., *Combating the COVID-19 epidemic: experiences from Vietnam.* International journal of environmental research and public health, 2020. **17**(9): p. 3125.
31. Aemro, A., et al., *Determinants of COVID-19 vaccine Hesitancy among Health Care Workers in Amhara region referral hospitals, Northwest Ethiopia: a cross-sectional study.* Epidemiology & Infection, 2021: p. 1-23.
32. Control, C.f.D. and Prevention, *COVID-19 Risks and Vaccine Information for Older Adults. Druid Hills: CDC. 2021.*
33. van der Valk, J., et al., *Case-control study of patient characteristics, knowledge of the COVID-19 disease, risk behaviour and mental state in patients visiting an emergency room with COVID-19 symptoms in the Netherlands.* PloS one, 2021. **16**(4): p. e0249847.

Table 1: Socio-demographic characteristics of study participants (N=418)

Variables	Category	Frequency	Percent (%)
Age	≤25	49	11.72
	26-30	234	55.98
	≥31	135	32.30
Sex	Female	129	30.86
	Male	289	69.14

Marital status	Single	194	46.41
	Married	224	53.59
Educational status	BSc and below	230	55.02
	MSc and above	188	44.98
Monthly income	<6990	27	6.46
	6991-12800	357	85.41
	≥12801	34	8.13
Family size	≤2	223	53.35
	3-4	128	30.62
	≥5	67	16.03
School-age children	No	286	68.42
	Yes	132	31.58

Table 2: COVID-19 related characteristics of the study participants (N=418)

Social isolation	No	152	36.36
	Yes	266	63.64
Undergone COVID test	No	172	41.15
	Yes	246	58.85
Confident in health care services	Not confident	194	46.41
	Confident	184	44.02
	Very confident	40	9.57
Information by health authorities	Clear	134	32.06
	Inconsistent	93	22.25
	Unclear	191	45.69
Measurements by Gov’t	Not very adequate	186	44.50
	Not adequate	132	31.58
	Adequate	100	23.92
Risk to get COVID-19 infection	Low	96	22.97
	Moderate	103	24.64
	High	219	52.39
Risk to sever COVID-19 disease	Moderate/high	115	27.51
	Low	223	53.35
	No/not sure	80	19.14
Households age >60 years	No	355	84.93
	Yes	63	15.07
Compliance to social isolation	No	152	36.36
	Yes	266	63.64
Suspected COVID-19	No	176	42.11

Diagnosis	Yes	242	57.89
Undergone COVID-19 test	No	172	41.15
	Yes	246	58.85
Perception of your health status	Good	183	43.78
	Very bad	7	1.67
	Very good	228	54.55
Autoimmune problem /taking steroid	No	402	96.17
	Yes	16	3.83
Will you take COVID-19 vaccine	No	192	45.93
	Yes	226	54.07
Confident in the current COVID-19 vaccine	Confident	80	19.14
	Not confident	100	23.92
	Not very confident	230	55.02
	Very confident	8	1.91

Table 3: Factors associated with adherence of COVID-19 mitigation measures among health care workers in referral hospitals of Amhara regional state of Ethiopia (N=418)

**Variables**	**Category**	**COR**	**AOR**	**P-value**	**95% CI**
Monthly income	≤6990	1	1	1	
	6991-12800	0.21*	0.29	0.105	(0.06-1.29)
	≥12801	0.18*	0.15	**0.041**	(0.02-0.92)
Hesitancy to COVID 19 vaccine	No	1	1	1	
	Yes	0.09*	0.10	**<0.001**	(0.04-0.25)
Age	<26	1	1	1	
	26-30	0.53*	0.09	**0.001**	(0.02-0.39)
	≥31	0.97	0.25	0.065	(0.05-1.08)
Marital status	single	1	1	1	
	Married	0.72*	1.33	0.422	(0.66-2.68)
Undergone COVID-19 19 test	Yes	8.28*	6.64	**<0.001**	(3.10-14.22)
	No	1	1	1	
Information health by authorities	clear	0.89	1.79	0.250	(0.66-4.88)
	Inconsistent	0.37*	0.41	0.071	(0.15-1.07)
	Unclear	1	1	1	
Measures by Gov't	Not very adequate	1	1	1	
	Not adequate	0.29*	0.66	0.299	(0.29-1.45)
	Adequate	1.28	4.60	**0.003**	(1.66-12.78)
Risk to get COVID-19 disease	low	1	1	1	
	Moderate	0.89	2.40	0.136	(0.75-7.61)
	High	3.09*	2.13	0.125	(0.81-5.60)
Fear to risk of sever COVID-19 disease	No risk	0.13*	0.16	**0.001**	(0.06-.46)
	Low risk	0.57*	0.45	10.07	(0.18-1.08)

	Moderate/high risk	1	1	1	
Household with age >60yrs	Yes	1.8 0*	7.94	**<0.001**	(3.14-20.04)
	No	1	1	1	
Suspected to COVID-19 infection	Yes	12.51*	5.74	**0.003**	(1.81-18.16)
	No	1	1	1	
Comorbidity	No	1	1	1	
	Yes	0.14	0.35	0.347	(0.04-3.08)
Confident on COVID-19 vaccine	Not confident	0.50	0.78	0.618	(0.29-2.07)
	Confident	1	1	1	

*=variables associated with the outcome variable at p-value<0.2, 1= reference category of the respected variable.

Figure

Click here to access/download;Figure;Fig 1.tif

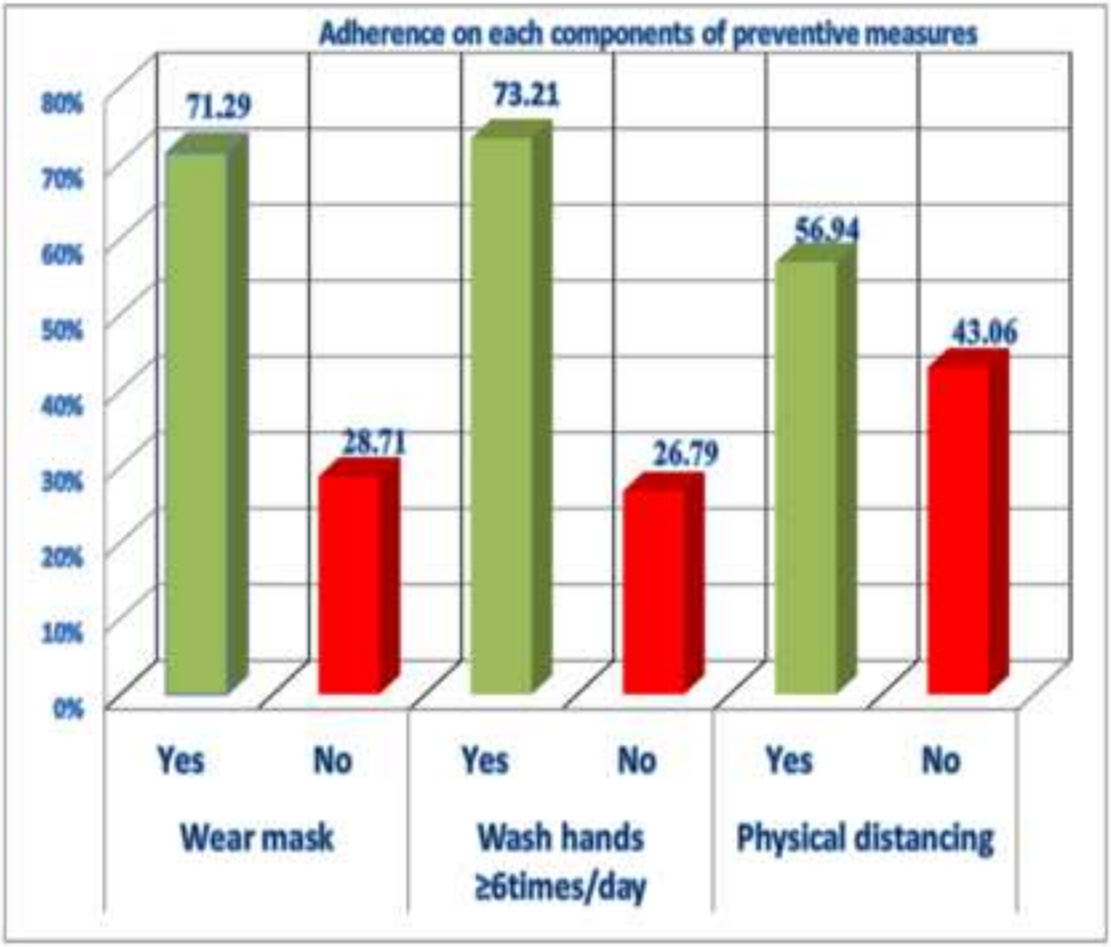

Supplement: Attachment — Submitted filename: PONE-D-21-38230 commented.pdf [file pone.0272570.s003.pdf]
