# Supplementary material for: Adherence to Covid-19 mitigation measures and its associated factors among health care workers at referral hospitals in Amhara regional state of Ethiopia
Source: PLoS One. 2022 Aug 5;17(8):e0272570. doi: 10.1371/journal.pone.0272570 (PMC9355263; doi:10.1371/journal.pone.0272570)
Supplement: Attachment — Submitted filename: PLOS point by point response .docx [file pone.0272570.s004.docx]

26/02/2022

Paavani Atluri,
PLOS ONE

**Dear Paavani Atluri,**

Subject: Submission of **revised manuscript entitled as “Adherence of Covid-19 mitigation measures and its associated factors among health care workers at Referral Hospitals in Amhara Regional state of Ethiopia” (PONE-D-21-38230).**

Thank you for email dated on January 21/2022 enclosing the Editorial member’s and the reviewer’s comments. We have carefully revised the manuscript and incorporated their comments accordingly. Our responses are given in point-by-point response below.

We hope the revised version is suitable for publication and look forward to hearing from you in due courses.

Sincerely

Beletech Fentie

University of Gondar, College of Medicine and health Sciences, School of Nursing, Department of pediatrics and child health nursing.

**Point by point responses to Editorial Board Member’s and Reviewers’ comments.**

**Title of paper: Adherence of Covid-19 mitigation measures and its associated factors among health care workers at Referral Hospitals in Amhara Regional state of Ethiopia**

**Editorial comments:**

1. Please ensure that your manuscript meets PLOS ONE's style requirements, including those for file naming

**Authors Response: Thank you very much for your constructive comments and suggestions.** We tried to incorporate your comments accordingly and we hope the manuscript meets PLOS ONE’s style requirements.

2. Please provide additional details regarding participant consent. In the ethics statement in the Methods and online submission information, please ensure that you have specified (1) whether consent was informed and (2) what type you obtained (for instance, written or verbal, and if verbal, how it was documented and witnessed). If your study included minors, state whether you obtained consent from parents or guardians. If the need for consent was waived by the ethics committee, please include this information.

**Authors’ Response: Thank you very much for your constructive comments.** Written informed consent was obtained from each participant using communication channels (telegram and email) and those who agreed to participate were included in the study and this information is provided in the **Ethics approval and consent to participate** section of the manuscript. This study did not include the minors.

3. We suggest you thoroughly copyedit your manuscript for language usage, spelling, and grammar. If you do not know anyone who can help you do this, you may wish to consider employing a professional scientific editing service.

**Authors’ Response: Thank you very much for your constructive comments.** We tried to address the comments in the manuscript**.** Since the authors are in low income country to cover the cost, online grammar checker was used to correct the spelling and grammar errors ([Grammarly.com)](https://www.googleadservices.com/pagead/aclk?sa=L&ai=DChcSEwjCoID-oZX2AhWK13cKHQ5YBP8YABAEGgJlZg&ohost=www.google.com&cid=CAESWuD2MsSyEdST_hGxOHfWNy8BM5xy5jOvD2anC4mpl1hW9DTZDTq_PQSiSUaNXKJpCKTSeHYOSW_ngofchtttF8Rx91uGkefUFRZ83FB_LK8M__1IlzSmHgUCzA&sig=AOD64_0IuiPdPk5j14aCosXpPlRB0gQsog&q&adurl&ved=2ahUKEwi76ff9oZX2AhWFxqQKHVLFA8cQ0Qx6BAgHEAE)

4. We note that you have indicated that data from this study are available upon request. PLOS only allows data to be available upon request if there are legal or ethical restrictions on sharing data publicly.

**Authors’ Response: Thank you very much for your comments.** We have uploaded the minimal anonymized data set necessary to replicate our study findings as Supporting Information file.

5. PLOS requires an ORCID ID for the corresponding author in Editorial Manager on papers submitted after December 6th, 2016.

**Authors’ Response:** Thank you very much for your comment. The corresponding author has validated her ORCID ID in Editorial Manager.

6. Your ethics statement should only appear in the Methods section of your manuscript. If your ethics statement is written in any section besides the Methods, please delete it from any other section.

**Authors’ Response:** Thank you very much for your comment. We deleted the ethics statement that was included other than the methods section in the manuscript.

7. Thank you for submitting the above manuscript to PLOS ONE. During our internal evaluation of the manuscript, we found significant text overlap between your submission and the following previously published works, some of which you are an author.

- <https://journals.plos.org/plosone/article?id=10.1371%2Fjournal.pone.0257373>

**Authors’ Response:** Thank you very much for your comments. We tried to revise the manuscript and rephrase the duplicated text and cite the sources. The published article you mentioned (<https://journals.plos.org/plosone/article?id=10.1371%2Fjournal.pone.0257373>) is conducted in the general community but the current study is conducted in health care providers which is different population in the general community(i.e The study populations of the already published article and the current manuscript is totally different). Therefore, we think there is no duplication.

8. Please review your reference list to ensure that it is complete and correct. If you have cited papers that have been retracted, please include the rationale for doing so in the manuscript text, or remove these references and replace them with relevant current references. Any changes to the reference list should be mentioned in the rebuttal letter that accompanies your revised manuscript. If you need to cite a retracted article, indicate the article’s retracted status in the References list and also include a citation and full reference for the retraction notice

**Authors’ Response:** Thank you very much for your comments. We have assessed the references as much we can and the references are correct and there is no retracted article cited.

**Reviewer #1:**

1. Since your study is a form of survey study, could you add study map?? to have more description for the reader?

**Author’s response:**

- Based on the comment, the authors incorporated a study map in the revised manuscript.

2. Correct place for ethics approval and consent to participate subtitle, and no need to write duplicate subtitle.

**Author’s response:**

- Thank you for the comments.
- Based on the comment, the authors removed the ethics section from the declaration part and only incorporated it in the method section.

3. No need to describe the study area / objective under discussion again!!

**Author’s response:**

- Thank you. The authors removed it in the revised manuscript.

4. Duplicate statements under abstract and conclusion.

**Author’s response:**

- Thank you for your concern. In the revised manuscript, the authors rephrase the idea in the abstract section.

**Reviewer #2**:

1. When you are writing the statement of the problem, it was nice if you put the paragraphs as follows; • Concise description of the problem (severity, which group is affected, the distribution of the problem, what contribute to the problem, the consequences of the problem. what policies, and strategies are in palace to combat the problem , what is known, what is not known, why you are interested in the topic ( what gaps exist).

**Authors Response: Thank you very much for your constructive comments and suggestions.** We tried to address your comments and suggestions accordingly in the manuscript.

2. Better to put in such way Binary logistic regression was be employed to identify factors associated with adherence of COVID-19 mitigation measures. Initially bivariate analysis was done and variables with p-value of below 0.2 were identified as candidate for multi-variable analysis. Then multi-variable analysis was done and adjusted odd ration was computed and interpreted. A p-value less than 0.05 is cut-off point for determining the significance of association. Result of the study was presented in text, table and graphs.

**Author’s response:** Based on the comments, the authors revised this paragraph under the subheading of the “Data processing and analysis” section of the manuscript.

**Reviewer #3:**

None

**Author’s response**: Reviewer 3 didn't have any comments to the authors regarding the manuscript.

**Reviewer #4:**

I appreciate the authors for doing a research on the current pandemic disease. But I have some concerns.

**Author’s response:**

•Thank you for your appreciation and positive feedback.

1. Abstract is OK.

**Author’s response**: Thank you.

2. Methods: Please provide further detail how Random selection was carried out to select study participants.

**Author’s response**: Thank you for your concern. Based on the comments, the authors incorporated the details of randomization in the revised manuscript.

3. Please try to provide the detail of the specific questions used to assess adherence level for all the three components.

**Authors Response:** Thank you very much for your constructive comments and suggestions.

We tried to address all the issues raised in operational definition part of the revised manuscript.

**4.** Discussion: The justification provided by the authors on the discrepancy between the studies conducted in other part of the same country may be due to the difference in tool used to assess adherence. Please provide a clear justification why you preferred using a tool with only three components to assess adherence level when others used different tool (discussion part second paragraph line 13-14)

**Authors Response:** Thank you very much for your constructive comments.

The previous studies used more adherence components since they were conducted in the initial phase of the pandemic (like there were lockdown, no public transportation, satay at home rules, mass gathering and etc. in the world). Since stay at home, restriction of public transportation, mass gathering and any lockdown are removed; we used the three major components used to prevent the COVID- 19 pandemic. The three components are also highly recommended by world health organization and many other health authorities and organization including Ethiopian ministry of health.

5. In discussion, the author only compared their finding with other studies on the adherence level and no comparisons were made with other studies for factors affecting adherence level.

**Authors’ Response:** Thank you very much for your constructive comments**.** In some extent, we tried to address the comment in the manuscript, but as our search we didn’t get any similar factors associated to adherence in other articles conducted in health care providers. That is why we left not discussed the factors variables. Instead, we tried to show scientific facts why these factors influence the adherence.

6. Discussion part should cover the interpretation of the finding, comparison with other studies, explanation for discrepancies if it exists, and the limitation of the study. The discussion part could be more than what the authors provided.

**Authors Response:** Thank you very much for your constructive comments**.** We tried to elaborate the discussion part as per your comment and suggestion

**Reviewer#5**

Dear authors, thank you for your effort, I have some comments and questions in your work
**Author’s response:** Thank you for your feedback.

**Abstract:**

1) The conclusion part of the abstract is not based on your finding, it’s a general kind of conclusion

**Author’s response:** Thank you. We revised and retyped it based on the findings of this study.

**Introduction:**

2) Your introduction is not well conceptualized, you did not show the gap for doing this research, you said "there is no paper locally" but there are number of papers on covid mitigation measures of health workers even in Ethiopia

**Authors Response:** Thank you very much for your constructive comment. We tried to address the comments in the manuscript. But still we couldn’t get any article published in the study setting among health care providers even in Ethiopia.

**Methods:**

3) You have defined perceived susceptibility and risk of getting disease as the same variables when they actually are very different variable

**Author’s response:**

According to the health belief model (HBM),

- Perceived susceptibility is defined as a person’s subjective perception about their chance or risk of getting a certain condition, in this case, COVID-19.
- This means the literal definition of “Perceived susceptibility” is “perceived risk of getting a disease”.
- That was why the authors used the phrase “Perceived susceptibility/risk of getting COVID-infection”, which is to mean “perceived susceptibility of getting COVID-infection” or “perceived risk of getting COVID-infection”.
- In short, based on the definition of HBM, the authors used these two terms interchangeably.
- But, to avoid ambiguity, the authors used only “perceived susceptibility” in the revised manuscript.

4) You measured perceived severity and risk of getting severe disease as the same variable when they actually are different variable

**Author’s response:**

- According to HBM, “perceived severity” refers to a person’s belief about the seriousness or severity of a disease.
- i.e. Perceived severity of COVID-19 infection = Perceived risk of developing sever COVID-19 infection.
- That was why the authors used the term “perceived severity/risk of developing the severe disease**”.**
- But, to avoid ambiguity, the authors used only “perceived severity” in the revised manuscript.

5) You measured perceptions categorically which has a lot of limitation, what's your base to categorize perception? I recommend you to treat perception items as continuous variable

**Author’s response:**

- Thank you for your recommendation. But, we assessed it by using the Likert scale, as other literature has used before. After that, re-categorization was considered in a few variables if the chi-square assumption was not fulfilled.

6) How do you measured vaccine hesitancy, COVID-19 mitigation measures, perceived susceptibility and perceived severity please attach the questionnaire I want to see the questionnaire

**Author’s response:**

- The way of measuring these variables was stated under the subheading “Operational Definition” of the method section.
- Based on your request, we attached the questionnaire during the re-submission of the revised manuscript as a “supplementary file” .

**Result:**

7) You have wrongly interpreted the odds ratios that are less than one

**Author’s response:**

We think the interpretations of the Odds rations are correct. For example:- AOR=0.15 means study participants adhered 15% to mitigation measures. In another way, it can be interpreted as study participants were 85% less likely adhere to mitigation measures of COVID-19. That is why we used one of the two ways of interpretations. If authors are mistaken, they can correct with reasonable justifications you will give us.
